# Toward High Carrier Mobility and Low Contact Resistance: Laser Cleaning of PMMA Residues on Graphene Surfaces

**DOI:** 10.1007/s40820-016-0093-5

**Published:** 2016-05-05

**Authors:** Yuehui Jia, Xin Gong, Pei Peng, Zidong Wang, Zhongzheng Tian, Liming Ren, Yunyi Fu, Han Zhang

**Affiliations:** 1 Materials Physics Laboratory, State Key Laboratory for Mesoscopic Physics, School of Physics, Peking University, Beijing, 100871 People’s Republic of China; 2 Key Laboratory of Microelectronic Devices and Circuits (MOE), Institute of Microelectronics, Peking University, Beijing, 100871 People’s Republic of China; 3 School of Electronic and Computer Engineering, Peking University Shenzhen Graduate School, Shenzhen, 518055 People’s Republic of China

**Keywords:** Graphene, PMMA residues, Laser exposure, Carrier mobility, Contact resistance

## Abstract

**Abstract:**

Poly(methyl methacrylate) (PMMA) is widely used for graphene transfer and device fabrication. However, it inevitably leaves a thin layer of polymer residues after acetone rinsing and leads to dramatic degradation of device performance. How to eliminate contamination and restore clean surfaces of graphene is still highly demanded. In this paper, we present a reliable and position-controllable method to remove the polymer residues on graphene films by laser exposure. Under proper laser conditions, PMMA residues can be substantially reduced without introducing defects to the underlying graphene. Furthermore, by applying this laser cleaning technique to the channel and contacts of graphene field-effect transistors (GFETs), higher carrier mobility as well as lower contact resistance can be realized. This work opens a way for probing intrinsic properties of contaminant-free graphene and fabricating high-performance GFETs with both clean channel and intimate graphene/metal contact.

**Graphical Abstract:**

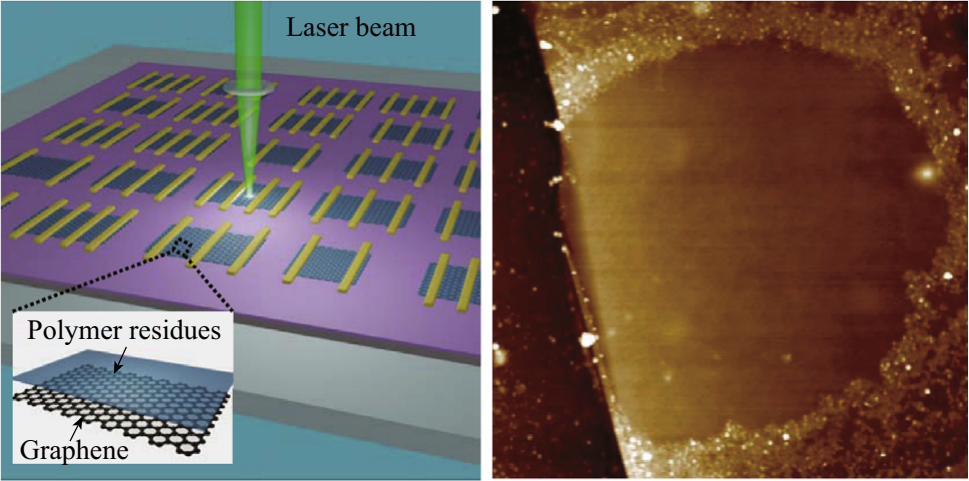

**Electronic supplementary material:**

The online version of this article (doi:10.1007/s40820-016-0093-5) contains supplementary material, which is available to authorized users.

## Introduction

Graphene, a single layer of *sp* ^2^ bonded carbon atoms, has attracted considerable interests for its intriguing physical properties such as high carrier mobility and thermal conductivity and held great promise for future integrated electronics [1–3]. Being a truly two-dimensional (2D) material, however, graphene is extremely sensitive to adsorbates and molecules in contact with its surface. The intrinsic properties of graphene are thus severely degraded because any surrounding medium may act as a dominant source of doping or scattering [4–6]. Unfortunately, contamination of graphene films with external molecules is inevitable in successive fabrication processes of devices, especially polymer residues.

To fabricate graphene field-effect transistors (GFETs), graphene grown by chemical vapor deposition (CVD) need to be transferred from a metal foil to an insulating substrate using a polymer such as poly(methyl methacrylate) (PMMA) as a support layer. PMMA is also commonly used as a mask material for electron beam lithography (EBL). Yet, a thin layer of PMMA residues (1–2 nm) after organic solvent (e.g., acetone) cleaning cannot be completely removed due to strong physical (van der Waals interactions) or chemical (covalent bonds formed between functional groups of PMMA and defect sites of graphene) adsorption effects [7].

Previous studies show that polymer residues left on graphene surfaces result in shift of the Fermi level and decrease of carrier mobility [4, 5]. Likewise, the polymer residues trapped at the interface of graphene/metal contact for GFETs fabricated in standard process considerably reduce graphene/metal interactions and lead to a broken ambipolar Fermi energy modulation and an increased contact resistance [8, 9]. To obtain a clean surface, graphene samples are empirically heated at 150–300 °C under Ar/H_2_ atmosphere or vacuum [7, 10, 11].

However, previous studies of transmission electron microscopy (TEM), X-ray photoelectron spectroscopy (XPS), Raman spectroscopy, and electrical measurements reveal that thermal annealing still cannot remove the polymer residues thoroughly. Furthermore, high-temperature heating process may intensify graphene/substrate and graphene/atmosphere interactions, causing graphene to be highly doped with severe mobility degradation [10–13]. In addition to thermal annealing, electric current-induced annealing [14, 15], wet chemical treatment [12, 16], plasma treatment [17, 18], and ultraviolet ozone treatment [19] have also been developed to address the problem of polymer residues. However, current-induced annealing is limited to GFETs with ready-made electrodes [14, 15]; wet chemical treatment by chloroform or formamide is often toxic and may bring in new species of contaminants [12, 16]; Ar or O_2_ plasma treatment is aggressive and needs to be operated with extremely low plasma density and delicate time control [17, 18]; ultraviolet ozone treatment has poor reproducibility and may induce serious oxidation of graphene under the same condition [19, 20].

Here we propose a new technique using a laser beam to eliminate polymer residues and recover clean graphene surfaces. Our laser cleaning technique, unlike previous methods, can be specially applied to targeted positions without introducing additional contaminants and defects. In the following contexts, detailed descriptions on laser cleaning process and optimization conditions are given. Then the laser cleaning technique is applied to GFETs, which shows that higher carrier mobility as well as lower contact resistance can be realized. Finally, mechanisms of laser cleaning are discussed in three ways: agglomeration, decomposition, and expulsion.

## Experimental Details

### Graphene Preparation and Measurements

Graphene was prepared using both mechanical exfoliation and CVD methods. The exfoliated graphene films were peeled off from natural flake graphite using an adhesive tape (3 M) at ambient conditions and transferred onto a heavily doped Si wafer coated with a 300-nm-thick thermally grown SiO_2_ layer. The CVD graphene films were grown on polycrystalline copper foil (25 μm thick, 99.8 %, Alfa Aesar) in a gas mixture of methane, hydrogen, and argon at 1000 °C. Then graphene films were transferred to a Si/SiO_2_ substrate. To describe the process of laser cleaning, both the exfoliated and CVD graphene films on Si/SiO_2_ substrate were intentionally spin-coated with a 270-nm-thick PMMA layer (Allresist AR-P 679.04), baked at 170 °C for 2 min, cooled to room temperature, and then placed in an acetone bath for 2 h to dissolve PMMA. The number of layers was first characterized by optical microscopy (Olympus BX51) and then confirmed by Raman spectroscopy (532 nm laser wavelength, 50× objective) and atomic force microscope (AFM, Bruker Dimension Icon) in air.

### GFET Fabrication and Electrical Measurements

Back-gated GFETs were fabricated in a top-down process. The graphene channels were patterned using e-beam lithography (EBL) followed by inductively coupled plasma reactive ion etching (ICP-RIE). The source (S) and drain (D) electrodes were fabricated by EBL, e-beam metal evaporation, and subsequent lift-off process. The exfoliated and CVD graphene films as the channel were contacted with Ti/Au (10/70 nm) and Pd/Au (20/60 nm), respectively. The structure of GFETs was inspected by optical microscope and AFM at tapping mode. All electrical measurements of GFETs were carried out on a probe station (Signatone WL-210E) using an Agilent B1500A semiconductor device analyzer under ambient conditions.

## Results and Discussion

**Fig. 1:**
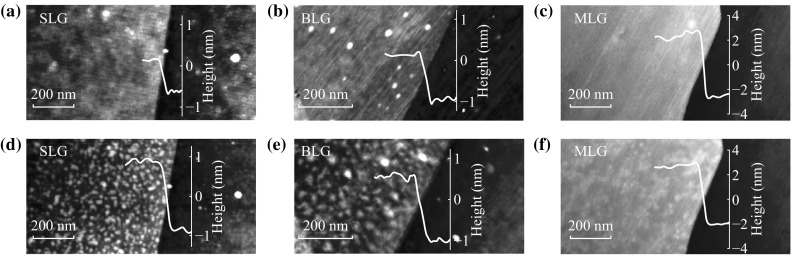
AFM topography images of mono-, bi-, and multilayer graphene, denoted as SLG, BLG, and MLG, respectively: before (**a**–**c**) and after PMMA contamination (**d**–**f**). Height profiles across the graphene edges are superimposed on the images

Figure 1 shows the AFM topography images of exfoliated graphene. The heights of the pristine single layer, bilayer, and multilayer graphene (denoted as SLG, BLG, and MLG) with respect to the SiO_2_ substrate were 0.695, 1.041, and 4.826 nm, respectively (Fig. 1a–c). The number of layers was confirmed by Raman spectroscopy, as shown in Fig. S1. The thickness of the measured monolayer graphene, larger than the interlayer spacing of graphite (0.335 nm), is attributed to a “dead” space between graphene and SiO_2_ [21]. However, after PMMA coating and acetone rinsing, the heights of the PMMA residue-adsorbed monolayer, bilayer, and multilayer graphene increased to 1.702, 1.648, and 5.236 nm, respectively, as shown in Fig. 1d–f. The graphene surfaces are covered by dense particle- or island-like PMMA residues. The thinner the graphene, the more the PMMA left on graphene. Compared with the Raman spectra of pristine graphene films, both the G band and 2D band for PMMA-contaminated exfoliated graphene samples show blue-shifts, especially the 2D band, indicating enhanced hole doping as well as intensified carrier scattering (Fig. S1) [7, 22, 23]. The root-mean-square (RMS) surface roughness *R*_q_ for the mono-, bi-, and multilayer graphene, averaged over 300 × 300 nm^2^ scan windows, increases from 0.151, 0.147, and 0.144 nm to 0.656, 0.552, and 0.368 nm, respectively. From single-layer graphene to multilayer graphene, *R*_q_ monotonously decreases because the short-range force between polymer residues and corrugated SiO_2_ substrate is gradually diminishing [24]. Similar tendency of *R*_q_ occurs for CVD graphene films as well (Fig. S2c). The PMMA residues on CVD graphene surfaces were introduced during the transfer process from Cu foil to SiO_2_ surface.

**Fig. 2:**
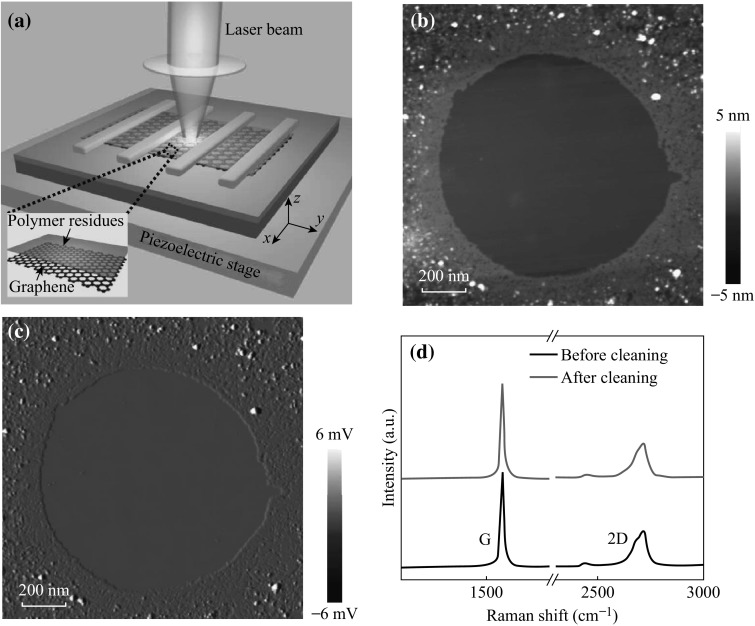
**a** Schematic of the laser cleaning process. AFM topography (**b**) and amplitude error (**c**) images of a PMMA residue-contaminated multilayer graphene after laser cleaning at 10 mW for 300 s. **d** Raman spectra of multilayer graphene before and after laser cleaning measured in situ at the central area in *panel* **b**

To remove the polymer residues, we simply made use of a home-built Raman system consisting of a 532 nm laser, a laser attenuator, a 50× objective, and a spectrometer (Princeton Instruments IsoPlane 160). Also a piezoelectric multi-axis stage with variable step size (minimum value: 0.1 μm) was mounted to locate or scan the graphene samples (Fig. 2a). The laser power was carefully calibrated and measured by an optical power meter (ThorlabsPM100D). For Raman spectroscopy, the laser power was kept below 2 mW to avoid laser-induced heating. Specifically, our laser cleaning approach using a visible laser from Raman system provides a unique benefit of real-time in situ Raman study of the effects of laser cleaning on graphene surface. Figure 2b shows the representative AFM topography image of a PMMA residue-adsorbed multilayer graphene after laser cleaning at 10 mW for 300 s at central part. Figure 2c shows the simultaneously captured amplitude error image, which is very helpful in visualizing fine details or subtle changes in surface topography [25]. A circular clean and smooth region is visible on the graphene surface with a diameter of ~1 μm, which is consistent with the size of laser spot of 10 mW beam. The RMS roughness *R*_q_ of the multilayer graphene after cleaning drops from 0.364 to 0.142 nm, approaching the *R*_q_ value of the pristine one, 0.144 nm. Moreover, the quality of graphene still remains high after laser cleaning, confirmed by the absence of the defect-induced D peak in the Raman spectrum as shown in Fig. 2d.

**Fig. 3:**
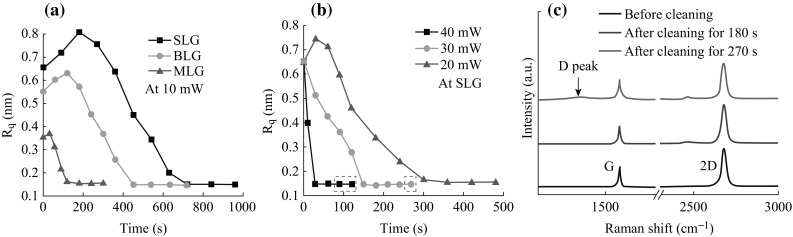
**a** Surface roughness *R*_q_ as a function of exposure time of mono-, bi-, and multilayer graphene with a fixed exposure power of 10 mW. **b** *R*_q_ as a function of exposure time of monolayer graphene with different exposure powers of 20, 30, and 40 mW. Dotted red boxes indicate the time regions when the disorder-induced Raman D peak occurs. **c** Raman spectra of the monolayer graphene before laser cleaning and after laser cleaning at 30 mW for 180 and 270 s, respectively. All *R*_q_ values are averaged over 300 × 300 nm^2^ scan windows

In an attempt to find optimum cleaning parameters, we systematically studied the effects of laser exposure power and time on contaminated graphene films with different numbers of layers. Figure 3a shows the surface roughness *R*_q_ as a function of exposure time of mono-, bi-, and multilayer graphene. Here, the exposure power was kept at a relatively low level of 10 mW. For thicker graphene, the resulting surface appears smoother with a lower value of *R*_q_, for it is less affected by the corrugated SiO_2_ substrate [24]. Meanwhile, it takes less time for thicker graphene to get rid of the PMMA residues. This may be explained by the different thermal performance for graphene with different numbers of layers. Comparing to mono- or bilayer graphene, multilayer graphene has lower thermal conductivity [26]. The laser-induced heat disperses into SiO_2_ substrate more slowly, leading to a higher surface temperature and thus a shorter cleaning time. With the time of laser exposure increasing, *R*_q_ first increases, then decreases, and finally stabilizes. The raise of *R*_q_ at the beginning may be attributed to agglomeration of polymer residues induced by laser heating. The mechanism of laser cleaning will be explained later in detail. We find that even up to 1000 s under mild laser exposure of 10 mW, no discernible Raman D peak occurs.

To save time, we increase the exposure power of laser. The dependence of *R*_q_ on exposure time for monolayer graphene with higher exposure powers of 20, 30, and 40 mW is shown in Fig. 3b. With exposure time increasing at the initial stage, *R*_q_ first increases and then decreases. With exposure time continuing to increase, *R*_q_ no longer decreases and restores closely to the value of its pristine state (~0.15 nm), which indicates that a nearly complete removal of polymer residues is achieved. The higher the exposure power, the faster the *R*_q_ decreases and saturates. However, it may induce defects at higher power (e.g., 30 and 40 mW). The dotted red boxes indicate the regions where the disorder-induced Raman D peak occurs. Figure 3c shows the Raman spectra of the monolayer graphene before and after laser cleaning with an exposure power of 30 mW for 180 and 270 s, respectively. The absence of the D peak around 1350 cm^−1^ indicates that there is no significant damage to the *sp* ^2^ hybridized carbon structure under a moderate exposure power of 30 mW for 180 s [27]. However, overexposure (e.g., for 270 s) will induce a few defects as evidenced by the emerging D peak. In the following electrical studies of graphene devices, we set the laser cleaning condition to be 30 mW (180 s)^−1^ for monolayer GFETs to realize fast, effective, and noninvasive removal of polymer residues.

This laser cleaning technique has also been applied to CVD mono-, bi-, and trilayer graphene, as shown in Fig. S3. The polymer residues left on CVD graphene samples are apparently removed, except at the ripples formed in wet transfer process where few residues may still remain due to increased chemical activity at these sites [28].

**Fig. 4:**
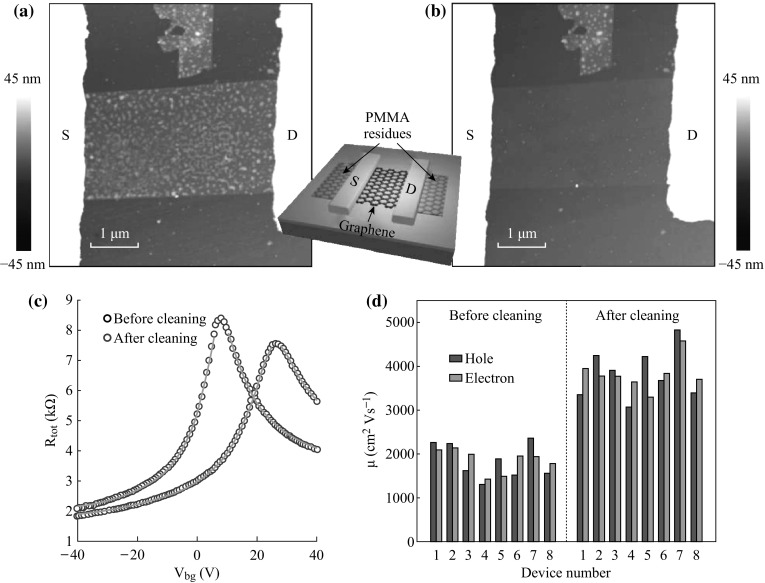
AFM topography images of a GFET before (**a**) and after (**b**) laser cleaning of the graphene channel. **c** Total resistance as a function of back-gate voltage of the GFET before and after cleaning of the graphene channel. *Solid red lines* are theoretical fits. **d** Histogram of electron (*red*) and hole (*blue*) mobility of GFETs. The *left* and *right* panels show the carrier mobility of GFETs before and after cleaning of the graphene channel, respectively. *Inset* schematic of a back-gated GFET. (Color figure online)

In the following, we demonstrate how this laser cleaning technique can be harnessed to remove PMMA contaminants from the graphene channel and graphene/metal contact in GFETs. For clarity, the effects of laser cleaning on graphene channel and contact were investigated independently. For the case of laser cleaning of the graphene channel, an exfoliated monolayer graphene was contacted by Ti/Au (10/70 nm) electrodes via EBL and lift-off metallization process to form a GFET. Figure 4a, b shows AFM topographies of the GFET before and after laser scanning over the whole graphene channel with a step size of 1 μm. A graphene fragment near the channel is also visible in these images, which can be used as a reference for comparison. Before cleaning, both the graphene channel and the graphene fragment are densely covered with PMMA particles, as shown in Fig. 4a. When comparing with the graphene fragment without laser exposure, the graphene channel appears much cleaner (Fig. 4b). Note that the graphene channel is so clean that it is hardly discernible from the SiO_2_ substrate. Figure 4c plots the corresponding total resistance as a function of back-gate voltage *V*_bg_ of the GFET before and after laser cleaning of the channel. Total resistance in our two-probe measurements is calculated from the transfer characteristics with *V*_bg_ swept from −40 to 40 V (*V*_ds_ = 0.1 V). The as-fabricated GFET exhibited a shift of the charge neutrality point (also referred to as the Dirac point *V*_D_) to 26.4 V, owing to the hole doping by polymer residues from EBL process [4, 5]. The electron and hole mobility of GFETs were extracted by fitting the n- and p-region of the ambipolar curves separately, according to the following equation [29, 30]:

1 $$R_{\text{total}} = R_{\text{contact}} + \frac{L}{{We\mu \sqrt {n_{ 0}^{2} + n^{2} } }},$$

where *R*_total_ is the total resistance; *R*_contact_ is the contact resistance; *L* and *W* are the channel length and width, respectively; *μ* is the carrier mobility; *n*_0_ is the carrier density at the Dirac point; *n* = *C*_bg_(*V*_bg_−*V*_D_) is the carrier density away from the Dirac point; and *C*_bg_ is the back-gate capacitance. There is a good agreement between measured data and theoretical fits as shown in Fig. 4c. For the as-fabricated GFET, electron and hole mobility were 2141 and 2230 cm^2^ (Vs)^−1^, respectively. The electron–hole asymmetry is generally attributed to charge transfer at the interface of graphene/metal contact, which forms p–n or p–p junctions for electron or hole cases and results in different transport properties [31, 32]. After laser cleaning, *V*_D_ shifted to 7.6 V, indicating reduction in hole doping. The near-zero yet non-zero *V*_D_ is attributed to oxygen or moisture adsorption from ambient atmosphere [24, 33, 34]. Removal of polymer residues causes a decrease in carrier scattering and thus an increase in both electron and hole mobility to 3770 and 4232 cm^2^ (Vs)^−1^, respectively. We measured eight GFETs before and after laser exposure. Histogram of electron (red) and hole (blue) mobility of these GFETs is shown in Fig. 4d, in which the left and right panels show the carrier mobility of GFETs before and after laser cleaning of the graphene channel, respectively. For GFETs with laser-cleaned channel, electron and hole mobility have been increased by a factor of 1.5–2.6. Enhancement of carrier mobility mainly originates from reduction of doping and scattering effects from extrinsic polymer residues [10, 11, 16].

The laser cleaning technique can also be used to remove the polymer residues from the contact regions of GFETs as defined by EBL prior to metal deposition. The previous thermal or current annealing methods, however, are not possible to remove the residual PMMA layer that is already covered by metal. To form intimate graphene/metal contact without polymer residues, generally there exist two kinds of processes in previous reports: the resist-free process and the resist-involved process. However, the resist-free process includes complex steps of non-polymer mask fabrication and alignment [8, 35]. The resist-involved process includes a global treatment by either oxygen plasma or ultraviolet ozone after contact lithography [18, 19]. As it is applied to the whole PMMA mask, resist deformation and thus pattern distortion may be caused. It is easy to remove the polymer residues on contact regions of GFETs using our laser cleaning technique.

**Fig. 5:**
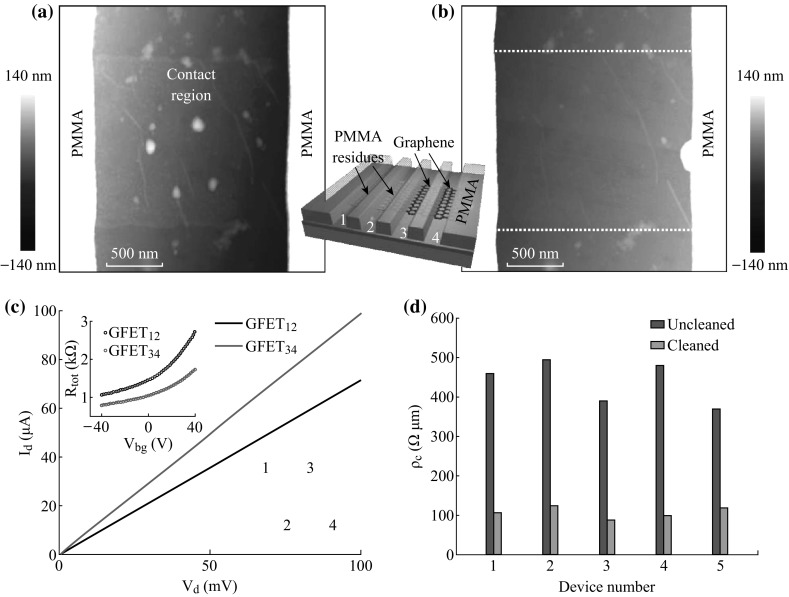
AFM topography images of a contact region before (**a**) and after (**b**) laser cleaning. *Middle inset* schematic of a PMMA mask pattern for fabrication of back-gated GFETs. **c** Output characteristics of two GFETs fabricated adjacently on a same graphene strip (*dotted white rectangle*) with identical geometry as shown in the *lower inset*. The electrodes are labeled as 1, 2, 3, and 4, respectively. The contact regions of GFET_12_ with electrodes 1 and 2 are uncleaned; and the contact regions of GFET_34_ with electrodes 3 and 4 are cleaned with laser exposure. *Upper inset* total resistance as a function of back-gate voltage (*V*_bg_) for GFET_12_ and GFET_34_, respectively. Solid red lines are theoretical fits. **d** Histogram of contact resistivity of five GFET groups with (*red*) and without (*blue*) being cleaned at the contact regions. (Color figure online)

CVD monolayer graphene was used to demonstrate the laser cleaning effects on contacts. The cleaning process at graphene contacts is as follows: first, a CVD graphene was transferred onto a SiO_2_ substrate by a PMMA layer and cut into a 1.4-μm-wide strip via EBL and inductively coupled plasma (ICP) etching. Then subsequently EBL was re-performed to define the electrode array. As shown in the middle inset of Fig. 5, the PMMA mask pattern for later deposition of metal electrodes has equivalent width and a spacing of 1.8 μm (labeled as 1, 2, 3, and 4, respectively). The laser beam was carefully focused on the opening windows of the PMMA mask. The representative AFM topography images for one of the contact regions before and after laser cleaning are shown in Fig. 5a, b, respectively. The contact region before cleaning was covered by dense PMMA residue particles, resulted from graphene transfer and lithography processes, while after laser cleaning at 30 mW for 180 s, the PMMA residues were effectively removed from the contact regions. The PMMA mask on both sides of the contact region shows no deformation. After this cleaning process, Pd/Au (20/60 nm) electrodes were directly evaporated onto the cleaned or uncleaned contact regions. And finally, the resist mask was dissolved by acetone in the lift-off process. No further laser cleaning of the graphene channels of the two GFETs was applied. Figure 5c shows the output characteristics of GFET_12_ and GFET_34_ with *V*_bg_ grounded. The device structure is shown in the lower inset of Fig. 5c. Comparing to GFET_12_ with uncleaned contacts, the *I*_d_–*V*_d_ curve of GFET_34_ with cleaned contacts exhibits a steeper slope indicating a lower total resistance. As the two GFETs are fabricated adjacently from a same graphene strip with nearly identical geometry, the reduction in contact resistance is supposed to be the main contributor to the reduction in total resistance. Contact resistance, extracted from total resistance (upper inset of Fig. 5c) by fitting the above Eq. 1, is 557.3 and 125.4 Ω for GFET_12_ and GFET_34_, respectively [36, 37]. As the fitted *R*_contact_ includes contributions from both source and drain, the contact resistivity (*ρ*_c_) is 390.1 and 87.8 Ω μm for uncleaned and cleaned GFETs, respectively. We measured five GFET groups with similar structure using the above local cleaning process. Figure 5d shows the histogram of contact resistivity of these GFET groups with uncleaned and cleaned contacts. The contact resistivity of GFETs with cleaned contacts has been decreased to 1/5–1/3 of those of GFETs with uncleaned contacts. The average contact resistivity of our GFET with cleaning contact (only 107 Ω μm) is much lower than the previously reported values (150–185 Ω μm for Pd contacts) [37, 38]. It shows that our laser cleaning technique is a reliable and efficient method to create low-resistance ohmic graphene/metal contact for high-speed GFETs.

It is worth noting that this laser cleaning technique may also be used in contact area with size smaller than 1 μm. The contact region shown in Fig. 5 is 1.8 μm wide. With a manual scanning, the PMMA mask on either side of the contact region was inevitably illuminated by the laser spot (~1.5 μm). However, as we can see from Fig. 5 that after laser cleaning, both sides show no deformation. Furthermore, no degeneration of the PMMA mask occurred because it was easily dissolved during lift-off process.

Besides PMMA residues, our laser cleaning is also effective for other polymer residues, such as novolak-based negative resist (AR-N 7520). After cleaning by 532 nm laser at 30 mW for 180 s, the residual negative-resist residues can also be completely removed, as shown in Fig. S4.

The mechanism of our laser cleaning process can be understood based on laser ablation phenomenon and PMMA behavior under laser exposure. Laser ablation (commonly by ultraviolet or near-infrared pulse lasers) has been widely used and thoroughly investigated for polymer micro-machining since the early 80s [39]. Here we give a qualitative interpretation for our laser cleaning of polymer residues on graphene surfaces in three ways: agglomeration, decomposition, and expulsion. At the initial stage of laser cleaning, the incoming photons penetrate and diffuse into the PMMA-contaminated graphene sample, raising the surface temperature and melting the polymer residues. The melted small PMMA particles, if originally densely packed, may merge into large PMMA droplets [40]. This is why we observe that the RMS surface roughness *R*_q_, as shown in Fig. 3a, b, is abnormally increased at the initial stage of laser exposure. With further laser irradiation, decomposition of polymer residues begins when the surface temperature approaches 230 °C [41]. In general, there are two models proposed to explain decomposition of PMMA by laser ablation: in the first model of thermochemical process, laser acts as a heating source and results in a solid–gas phase transition, prevailing in near-infrared lasers [42, 43]; in the second model of photochemical process, high-energy photons directly break the main-chain bonds, dominating in ultraviolet lasers [44, 45]. Previous studies show that decomposition of PMMA includes main depolymerization process into monomers (at least 80 % of the mass loss) and other secondary processes into low-molecular-weight gases (e.g., H_2_, CO, CO_2_, CH_4_, C_2_H_4_) in trace amounts [37, 46, 47]. As shown in the AFM images in Figs. 2, 3, 4, and 5 (also in Supporting Information), the PMMA residues are obviously removed from graphene surfaces after laser illumination. We thus speculate that both thermochemical and photochemical processes may potentially be possible to account for decomposition of PMMA in our continuous-wave visible laser cases.

**Fig. 6:**
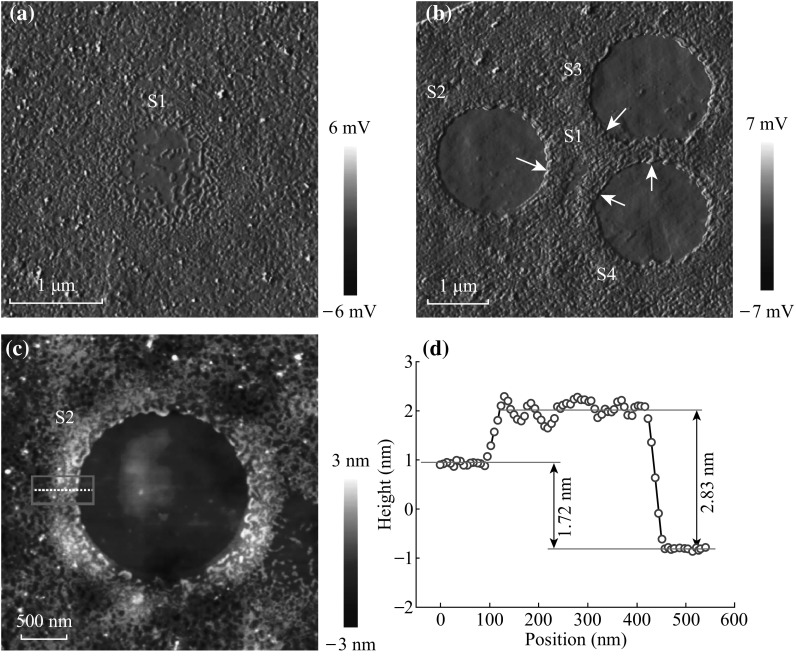
Four spots on a multilayer graphene sequentially cleaned with laser exposure. **a** AFM amplitude error image of a lightly cleaned spot at 10 mW for 60 s (denoted as S1) and **b** at 30 mW for 180 s, marked as S2, S3, and S4 according to the laser irradiation order. *White arrows* indicate the directions of expulsion of PMMA residues. **c** AFM topography image of S2. **d** Height profile along the *white dotted line*, averaged over the *red rectangular box* in **c**. (Color figure online)

On the other hand, during photon absorption, polymer residues not only gain energy but also momentum. Driven by monomer vapor pressure as well as laser light pressure, expulsion of liquid PMMA particles or even ejection of solid PMMA fragments occurs, thus facilitating removal of polymer residues [42, 48, 49]. Figure 6a shows a lightly cleaned spot denoted as S1 after 10 mW laser exposure for a short time of 60 s. As the laser beam presents a Gaussian distribution [50], where energy peaks in its center and drops smoothly to its periphery, the center of the radiated spot gains more energy with respect to the periphery. As shown in this image, the hotter center appears cleaner with very few PMMA particles left, while in the cooler periphery, besides decomposition, thermal expansion and migration of PMMA particles induce further agglomeration to PMMA droplets. Figure 6b shows the subsequently cleaned spots (near S1 position) after 30 mW laser exposure for 180 s, sequentially denoted as S2, S3, and S4 according to the laser irradiation order. As we can see, the originally circular S1 is severely compressed by S2 and S4. And the lower edge of S2 is also pushed upwards by S3. All phenomena reveal the effects of expulsion by intense pressure. Figure 6c shows the magnified height image of S2, of which the immediate edge region is highest. Specifically, height profile averaged over the red dotted box along the white dotted line is shown in Fig. 6d. The height difference between the outermost contaminated region and the center cleaned region is measured to be 1.72 nm, while the immediate edge region shows an average height difference of 2.83 nm. As described previously, accumulation of the polymer residues at the edges is partly attributed to expulsion of the liquid particles and partly due to the re-deposition of the vapor monomers or PMMA residues.

## Conclusions

In summary, we have proposed a facile and reliable technique to remove polymer residues on graphene surfaces without generating defects using a visible laser from Raman system. After laser cleaning of the channel in GFETs, carrier mobility has been improved by a factor of 1.5–2.6. Moreover, this technique can be particularly applied to the contact regions as defined by EBL prior to metal deposition to eliminate the polymer residues, which is impossible by previous annealing methods. The contact resistivity of GFETs with cleaning at contacts can be reduced to 1/5–1/3 of those of GFETs without cleaning. This work provides an efficient route to get access to intrinsic properties of polymer residue-free graphene and fabricate high-speed GFETs with high carrier mobility and low contact resistance.

## Electronic supplementary material

Below is the link to the electronic supplementary material.

Supplementary material 1 (PDF 805 kb)
