# Supplementary material for: Toward High Carrier Mobility and Low Contact Resistance: Laser Cleaning of PMMA Residues on Graphene Surfaces
Source: Nanomicro Lett. 2016 May 5;8(4):336–46. doi: 10.1007/s40820-016-0093-5 (PMC6223693; doi:10.1007/s40820-016-0093-5)
Supplement: Supplementary file 1 — Supplementary material 1 (PDF 805 kb) [file 40820_2016_93_MOESM1_ESM.pdf]

Supporting Information for

Towards High Carrier Mobility and Low Contact Resistance: Laser Cleaning of PMMA Residues on Graphene Surfaces

Yuehui Jia[1,2], Xin Gong[3], Pei Peng[2], Zidong Wang[2], Zhongzheng Tian[2], Liming Ren[2], Yunyi Fu[2,*], Han Zhang[1]

[1] Materials Physics Laboratory, State Key Laboratory for Mesoscopic Physics, School of Physics,

[2] Key Laboratory of Microelectronic Devices and Circuits (MOE), Institute of Microelectronics, Peking University, Beijing 100871, People's Republic of China

[3] School of Electronic and Computer Engineering, Peking University Shenzhen Graduate School, Shenzhen 518055, People's Republic of China

*Corresponding author. E-mail: yyfu@pku.edu.cn

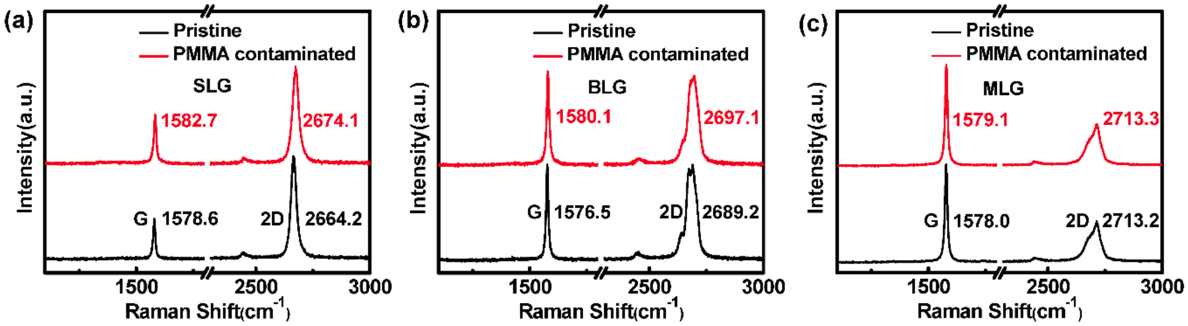

Fig. S1

Fig. S1 Raman spectra of the pristine and PMMA contaminated monolayer (**a**), bilayer (**b**) and multilayer (**c**) graphene prepared by mechanical exfoliation, corresponding to Figure 1 in the main text. Peak positions of the pristine (black) and PMMA contaminated (red) graphene samples are marked aside. Both the G band and 2D band of the graphene exhibit blueshifts, indicating enhanced hole doping as well as intensified carrier scattering.

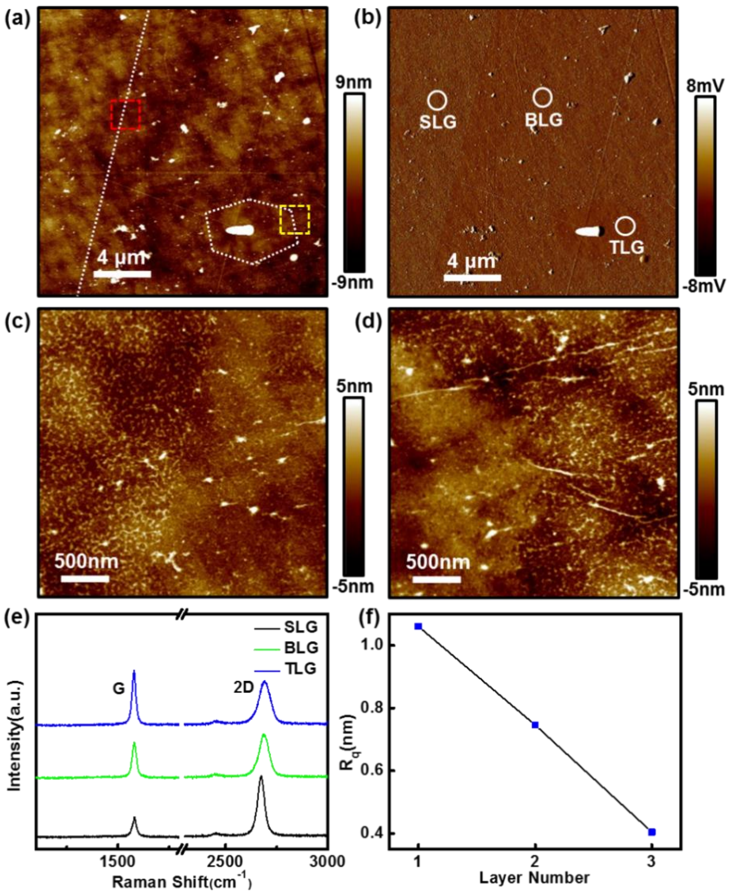

Fig. S2

Fig. S2 AFM topography image (**a**) and corresponding amplitude error image (**b**) of a CVD graphene on SiO_2 substrate. Regions of mono-, bi- and trilayer graphene are marked as SLG, BLG and TLG, respectively. White dotted lines indicate the boundaries of the regions. Magnified topography images of the boundary zones corresponding to the red dotted square (**c**) and yellow dotted square (**d**), respectively. Graphene surface has already been contaminated with polymer residues during the wet-transfer process. The thicker the graphene, the less PMMA particles left. (**e**) Raman spectra of the CVD mono-, bi- and tri-layer graphene, respectively, measured from the marked circles in (b). (**f**) Surface roughness Rq as a function of the number of graphene layers. All Rq values are averaged over 300×300 nm^2 scan windows. R_q monotonically decreases with increasing number of graphene layers.

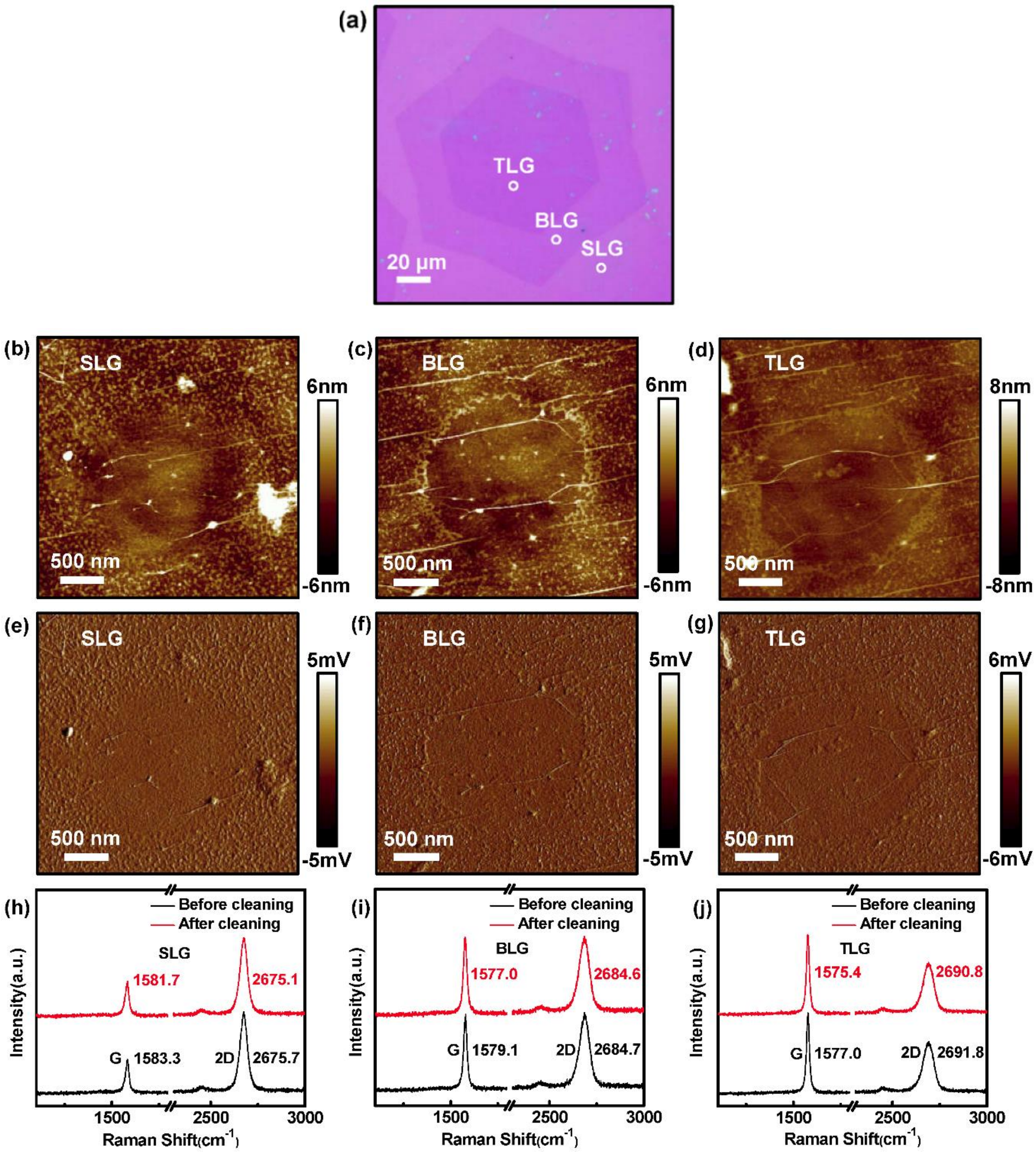

Fig. S3

Fig. S3 (**a**) Optical image of a CVD-grown graphene on SiO_2 substrate. AFM topography (**b-d**) and amplitude error (**e-g**) images of mono-, bi- and trilayer graphene after laser cleaning at the regions marked by white circles in (a). (**h-g**) Raman spectra of mono-, bi- and trilayer graphene before and after laser cleaning, respectively. Peak positions of the graphene before (black) and after (red) laser cleaning are marked aside

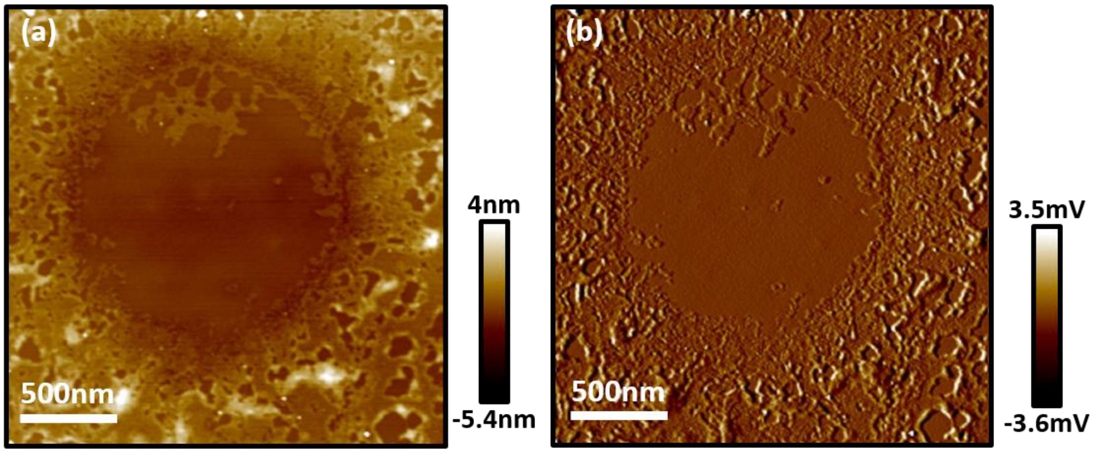

Fig. S4

Fig. S4 AFM topography (**a**) and amplitude error (**b**) images of a novolak-based negative resist residues on multilayer graphene after exposure to a 532 nm laser at 30 mW for 180 s in the center area
